# Understanding death wishes in later life: a narrative review

**DOI:** 10.3389/fpsyt.2026.1791344

**Published:** 2026-06-19

**Authors:** Richard C. Oude Voshaar, Radboud M. Marijnissen

**Affiliations:** Department of Psychiatry, University of Groningen, University Medical Center Groningen, Groningen, Netherlands

**Keywords:** death wishes, existential suffering, geriatric psychiatry, medical assistance in dying (maid), narrative identity, social connectedness

## Abstract

**Background:**

Death wishes and end-of-life legislation are increasingly relevant in ageing societies, where demographic shifts and evolving legal frameworks appear to raise complex ethical and clinical challenges.

**Summary:**

Wishes to die among older adults are complex and not limited to mental illness or terminal illness. Although depression and demoralization are common, evidence suggests that many older individuals experience these wishes in the absence of psychiatric disorders. These experiences often reflect an existential response to cumulative losses in autonomy, identity, and social embeddedness. This review organizes existing literature using a multilevel explanatory framework. At the psychological–existential and identity level, death wishes are associated with depression, demoralization, loss of meaning, and narrative disruption. At the social–relational level, loneliness, perceived burdensomeness, and social disconnection play a central role. At the structural–cultural level, ageism, societal narratives of dependency, and broader cultural meanings of ageing contribute to the emergence and interpretation of death wishes. At the legal–ethical level, medical assistance in dying (MAiD) frameworks shape how autonomy, suffering, and legitimacy of death wishes are understood in different jurisdictions. Across these levels, death wishes appear to arise from interacting psychological, relational, and societal processes rather than from a single underlying cause. Qualitative studies further highlight the deeply personal nature of these experiences, often linked to feelings of being a burden, narrative closure, or perceived loss of future meaning. Clinically, evidence indicates that a purely psychiatric or autonomy-driven approach is insufficient. Instead, effective responses require narrative competence, existential sensitivity, and awareness of contextual influences. This review synthesizes these findings into an integrated conceptual framework and outlines key implications for clinical practice in geriatrics, psychiatry, and palliative care.

**Key message:**

This review argues for a multidimensional approach that recognizes death wishes not only as clinical or legal phenomena, but as deeply human expressions of suffering, identity, and relationality in the context of ageing.

## Introduction

Amid global ageing, the phenomenon of death wishes among older adults has gained increasing attention across clinical, ethical, and societal domains. Whereas academic discourse has traditionally centered on suicidality and depressive symptomatology in later life, emerging research has elucidated a more complex and multifaceted conceptualization of death wishes in older adults without terminally illness or psychiatric disorders (1, 2). In particular, the emergence of legal and ethical debates on physician-assisted dying for those experiencing a “completed life”—notably in the Netherlands and Belgium—has foregrounded the urgency of clarifying the conceptual boundaries and empirical correlates of this phenomenon (3, 4).

A key challenge in this field lies in the heterogeneity of terminology and operational definitions. Death wishes may encompass a broad continuum of experiences, ranging from transient, passive thoughts about the end of life to persistent, well-considered requests for MAID (5). Critically, not all individuals who report such wishes meet criteria for major psychiatric disorders, nor do they invariably express suicidal ideation as traditionally defined (6, 7). This observation necessitates a conceptual distinction between psychopathology-driven suicidal ideation and existentially or contextually rooted death wishes. In this review, death wishes are conceptualized as a spectrum ranging from a diminished will to live and passive wishes for death, to more persistent and articulated wishes to die, and in some cases explicit requests for assisted dying Importantly, these experiences are not always clearly separable from suicidal ideation and may partially overlap with it, which itself typically involves a more direct intent to actively end one’s life and is more often associated with acute psychopathology (5, 6). For classification of death wishes see **Table 1** (7–11).

The current narrative review aims to synthesize recent empirical literature and conceptual developments concerning death wishes in later life, including 1) epidemiology, 2) phenomenological typologies and experiential aspects, 3) psychological and psychiatric correlates, 4) social, existential, and contextual determinants, 5) ethical and legal challenges related to medical assistance in dying and other end-of-life decision-making practices. To organize this complexity, we adopt a multilevel framework distinguishing psychological–existential and identity-related processes, social and relational determinants, structural and cultural influences, and legal–ethical dimensions.

## Methods

We conducted a narrative review with a multilevel conceptual approach, integrating empirical, qualitative, and theoretical literature on death wishes in older adults. PubMed, PsycINFO, and Google Scholar were searched for English-language studies published between 1990 and 2025 using terms such as “death wishes,” “tired of life,” “elderly suicide ideation,” “completed life,” “MAID,” “older and “elderly” and “existential suffering.” Both authors (ROV; RM) independently screened titles, abstracts, and full texts. Inclusion criteria were studies on adults aged 55+, conceptual or ethical analyses, and interventions relevant to existential or psychosocial dimensions; studies limited to terminally ill younger populations or non-English publications were excluded. Relevant literature was synthesized narratively to capture epidemiological, psychological, social, existential, ethical, and legal dimensions, with attention to conceptual models and clinical implications.

### Epidemiology

Death wishes are often complex and may range from a general desire to die to an active wish to end one’s life. Importantly, estimates of prevalence vary substantially across studies, largely reflecting heterogeneity in operational definitions (e.g., wish to live vs. wish to die vs. suicidal ideation), measurement instruments, and recall timeframes (e.g., lifetime, past year, past week), rather than true differences in population rates. For example, among 1,794 older adults (aged 58–98) from the general population in the Netherlands participating in the Longitudinal Aging Study Amsterdam (LASA), 18.7% reported having ever experienced a wish to die (12). When limited to the past week, 1.2% reported a diminished will to live without a wish to die, 1.4% reported a weak wish to die, and 0.8% reported a strong wish to die (12). Recognizing such nuances is essential in both clinical practice and research. Irrespective of the exact definition, however, death wishes are present in a substantial proportion of the older population, with prevalence estimates ranging from 2.3% to 11.6% that center around 3 – 4% (see **Table 1**). One population-based study reported a very low prevalence outside this range, i.e. 1.25% (13). This low prevalence rate can be explained by the fact that the denominator was restricted to people who considered themselves as not having a severe illness (13). Interestingly, all studies thus far have been conducted in Western societies (Australia, New Zealand, Netherlands, USA, Ireland, Sweden, Germany, Switzerland).

**Table 1:** Classification of death wishes.

Term	Definition	References
Persistent death wish	A persistent wish to die that has been present for a year or longer. Persistence refers primarily to the duration of the death wish, not its intensity.	Paykel et al., 1974 (7)
Latent / passive death wish	A desire to die without concrete plans, actions, or steps to actually hasten death. There are no serious considerations to end one’s life.	Van der Heide et al., 2014 (8); van Orden et al., 2015 (9)
Active death wish	A wish to die accompanied by concrete planning and/or actions related to the death wish and/or hastening death. This may take various forms, such as exploring options by seeking information or discussing the wish with others, drafting an advance directive, discontinuing medication, making plans to end one’s life, requesting assistance in dying, or submitting a euthanasia request. It may also include seriously considering ending one’s life within the past year.	Van der Heide et al., 2014 (8); van Orden et al., 2015 (9)
Wish for termination of one’s life	The wish to actually end one’s life. This closely relates to an active death wish, though the two are not identical. Individuals may be actively engaged with their death wish without yet being willing to take the ultimate step.	Ohnsorge et al., 2014a, 2014b (10, 11).

As shown in **Table 2** (12–26), the prevalence of death wishes among specific subgroups of patients ranges between 6.1 and 12.9% and centers around 8%; a doubling compared to the general population.

**Table 2:** Prevalence of death wishes in the general population and within specific patient groups.

Study	Sample Size (n)	Age range (years)	Prevalence of Death Wishes	Definition/Measurement of Death Wishes
Population-based samples				
Jorm et al., 1995 (14)	923	70+	2.3% repeated wish to die	Epidemiological survey questions on a repeated wish to die in past two weeks.
Skoog et al., 1996 (15)	345	85+	4.0% mentally healthy 27.5% with mental disorders	Psychiatric examination
Forsell et al., 2000 (16)	1,099	75+	11.6% death wishes (baseline) 8.9% death wishes (follow-up)	Structured psychiatric interviews
Rurup et al., 2011a (12)	1,794	58-98	3.4% current wish	Interview asking for current wish to die and/or weakened wish to live
Hartog et al., 2020 (17)	21,294	55+ (no severe illness)	1.25% persistent death wish	Survey assessing persistent death wishes without severe illness
Briggs et al., 2021 (18)	8,174	50+	3.5% wish to die	Self-report question “In the last month, have you felt that you would rather be dead?”
Zomers et al., 2021 (19)	3,141	75+	2.07% persistent wish	Survey assessing persistent death wishes without severe illness
Ward et al., 2024 (20)	6,915	50+	3.6% wish to die	Self-report question “In the last month, have you felt that you would rather be dead?”
Clinical samples				
Chochinov et al., 1995 (13)	200	Terminally ill (mean 70.9; range 31-94 years)	44.5% occasional 8.5% serious	Semi-structured interviews assessing desire for death and depressive episodes
Kim et al., 2006 (21)	355	Primary care patients (65+)	9.7% thoughts of death 6.1% wish to die	Standard questions of the Composite International Diagnostic Interview (CIDI) Depression section
Rosenfeld et al., 2006 (22)	372	Advanced AIDS (mean 44.4; range 23-75)	4.6% clinician rated 8.3% self-report	Clinician-rated and self-report measures of desire for hastened death
Cheung et al., 2017 (23)	35,734	Older adults assessed for home support and long-term care (65+)	9.5% death wishes	interRAI Home Care assessment data
Bornet et al., 2020 (24)	232	Hospitalized patients at internal ward (65+)	8.6% wish to die	Schedule of Attitudes toward Hastened Death-senior and the Categories of Attitudes toward Death Occurrence scales
Dürst et al., 2020 (25)	101	Geriatric rehabilitation patients (65+)	12.9% significant wish to die	SAHD-Senior and CADO instruments
Vehling et al., 2021 (26)	2,141	Cancer patients (range 18-75)	6.9% thoughts of death without suicidal ideation 4.1% suicidal ideation	Composite International Diagnostic Interview-Oncology (CIDI-O)

MNC, mononuclear; PEG-rhG-CSF, pegylated recombinant human granulocyte colony stimulating factor; rhG-CSF, recombinant human granulocyte colony-stimulating factor; VP-16, etoposide.

### Phenomenology of death wishes

Several qualitative studies underscore the experiential diversity of death wishes in later life, challenging monolithic or symptom-based interpretations. One qualitative interview study delineated a typology of metaphoric self-representations among older adults with a persistent wish to die, including the “wreck”, “prisoner”, and “burden” (27). These metaphors reveal deeply embodied feelings of loss of dignity, autonomy, and social embeddedness—experiences which are not adequately captured by standard psychiatric nosology.

Empirical research has sought to classify death wishes according to multiple dimensions, including intentionality, agency, temporal continuity, and their association with suffering. For instance, Dransart et al. (2021) proposed a typology that distinguishes 1) latent wishes to die (passive, often unarticulated), 2) expressed wishes (verbalized but not acted upon), and 3) requests for assisted dying (formally initiated under legal frameworks) (5). These distinctions echo earlier conceptual models by Rurup et al. (2011), who identified both emotional and rational underpinnings of death wishes, with the latter frequently devoid of clinical depression (1). Their findings also resonate with Ayalon’s (2010) observation that death wishes may function as a form of existential protest against perceived futility, social exclusion, or narrative disintegration (28). Further empirical support comes from the work of Fässberg et al. (2014), who found that death wishes were often associated with loneliness and functional disability, independent of depressive symptom severity (6). These findings are consistent with Joiner’s Interpersonal Theory of Suicide (29), which posits that a disturbed sense of belonging and perceived burden are primary psychological drivers of a death wish.

Critically, death wishes should not be interpreted as static or terminal expressions. Multiple studies report substantial temporal fluctuation in the intensity and presence of death wishes, often modulated by changes in physical health, social connectedness, and meaning-making processes (30). This dynamic suggests that in many cases death wishes may be better conceptualized as relational and reversible states, rather than as fixed endpoints in a life course. The phenomenology of death wishes thus reveals a complex interplay between one’s personal history, embodied suffering, and social-cultural narratives. Rather than pathologizing these expressions, a hermeneutic approach—emphasizing narrative identity, dignity, and existential meaning—may offer a more ethically adequate and clinically informative framework (31).

### Psychological correlates

The public debate surrounding medical assistance in dying (MAID) in the context of an ageing population, has revitalized research into the psychological underpinnings of death wishes among older adults. Whereas earlier studies often conceptualized such wishes as direct expressions of depressive disorders (32), more recent evidence points to a more differentiated understanding, encompassing not only depression, but also demoralization, identity disintegration, and existential despair.

#### Depression

Depression is undeniably one of the most frequently cited correlates of death wishes in older populations. Several epidemiological studies demonstrate a robust association between depressive symptomatology and both passive and active wishes to die [e.g. 6]. These findings are in line with earlier clinical literature, which identified hopelessness as a critical mediator between depression and suicidal ideation (33).

However, the explanatory power of depression alone appears limited. In a qualitative study among Dutch older adults with an expressed death wish identified in two large population-based cohort studies, only three out of the 31 participants had a history of recurrent depression (1). Even among older adults with severe psychiatric disorders, death wishes may emerge beyond psychopathology. In a clinical cohort of 1,784 individuals aged 60 years and older referred for specialized mental health care, a total of 302 (16.9%) patients expressed a death wish. Considering one’s life completed contributed independently to death wishes in this population, beyond age, suicidality, and severity of psychopathology (34). Existential concerns, such as the loss of autonomy, narrative coherence, and a sense of being meaningful, often underlie death wishes in the absence of psychiatric morbidity (35).

The concept of demoralization may offer a more accurate perspective for understanding these experiences. Defined as a state of existential distress characterized by helplessness, hopelessness, and a sense of failure (36), demoralization has been shown to predict suicidal ideation independently of depression severity. While not routinely assessed in geriatric psychiatry, its relevance is increasingly recognized in the palliative care literature [e.g. 37] and may also be relevant to understand persistent death wishes among non-depressed older adults.

#### Identity disruption and narrative collapse

Another recurring psychological theme is the loss of personal identity and the fragmentation of one’s life narrative. Within a phenomenological framework, older adults with a persistent death wish often describe that they no longer experience themselves as the person they once were (30). This loss of identity is often accompanied by functional decline, loss of independence, and social invisibility, all of which undermine one’s sense of agency and continuity over time. In this context, a death wish may not be a symptom to be “treated”, but rather a signal of profound existential disorientation. This view is supported by qualitative studies in which older adults describe their death wish not in terms of psychopathology, but as a rational endpoint to a life devoid of meaning, coherence, or future prospects (1, 29).

Such findings also relate to the developmental theory of Erikson, in particular the final psychosocial stage of ego integrity versus despair (38). When individuals fail to integrate past experiences into a meaningful whole, or when future goals are unattainable, despair may ensue—potentially culminating in a wish to die. Yet, many of these wishes reflect ambivalence, and may contain latent yearnings for change, connection, or relief (28).

#### Psychological models beyond psychiatry

It is increasingly recognized that psychological frameworks from outside conventional psychiatry can enrich our understanding of death wishes. The Interpersonal Theory of Suicide (29) postulates that two key constructs—perceived burdensomeness and thwarted belongingness—are critical in the emergence of suicidality. These constructs have been validated in older populations and may help explain death wishes in the context of relational and social breakdown, rather than individual psychopathology (39, 40). Similarly, meaning-centered approaches, derived from existential psychology emphasize the protective role of perceived purpose and meaning-in-life (41). Recent studies have found that low levels of meaning-in-life and physical frailty are strongly associated with death wishes in late life, independently of mood disorders (42, 43).

Taken together, the psychological factor behind death wishes in older adults are multifactorial and often extend beyond the boundaries of clinical depression. Future research should integrate psychiatric, existential, and identity-related dimensions to more adequately capture the complexity of this phenomenon.

### Social, existential, and contextual factors

Beyond individual psychological states, death wishes among older adults are deeply embedded in social, existential, and cultural contexts. A growing body of qualitative and mixed-method research demonstrates that these wishes may emerge in response to perceived social disconnection, a lack of meaningful roles, and a diminishing sense of existential value.

#### Loneliness, social isolation, and exclusion

Among the most consistently identified social correlates of death wishes is loneliness—both emotional and existential. Older individuals who wish to die often articulate a profound sense of disconnection, not merely from others, but from the social world as a meaningful structure (5, 12). This aligns with narrative analyses, which show how death wishes are frequently framed within experiences of invisibility, redundancy, and perceived social irrelevance (44). These subjective experiences are substantiated by epidemiological data showing that perceived loneliness was a stronger predictor of death wishes than depressive symptoms or chronic illness (6). Similarly, social isolation, particularly loss of emotionally significant ties, was found to mediate the relationship between functional impairment and death-related ideation (29). Importantly, these findings necessitate a move beyond the dichotomy of living alone versus being socially embedded. Several authors have highlighted that death wishes can occur even in socially “connected” individuals, when the quality of relationships is poor, characterized by dependency, asymmetry, or loss of reciprocity (45). In such cases, older persons may feel like a burden, reinforcing a wish to die not out of despair per se, but as a perceived act of altruistic withdrawal.

#### Loss of meaning and existential disintegration

Parallel to social marginalization, many death wishes appear rooted in what has been termed existential vacuum—a state marked by the absence of meaning, purpose, or future orientation (42). Older adults with a wish to die often perceive their life as a “closed book”, in which all meaningful chapters have been concluded (17). This is particularly pronounced in individuals who have outlived significant others, or who have experienced irreversible functional or cognitive decline. Such perceptions resonate with Eriksonian developmental theory, particularly the conflict between ego integrity and despair. In cases where life review processes result in regret, resentment, or an inability to find coherence in one’s life story, the existential ground for continued living may erode (21). Existential loss is further compounded by ageism—both societal and internalized. A recurring theme in qualitative studies is the perception of being “no longer needed”, “taking up space”, or “consuming resources” (27). This internalization of societal devaluation may reinforce feelings of worthlessness and validate death wishes in the absence of overt pathology.

#### Cultural and structural contexts

Finally, death wishes need to be understood within broader cultural and policy frameworks. In countries where MAID is legalized, public debate may inadvertently influence how older adults conceptualize their worth and agency by providing socially endorsed narratives about autonomy, independence, and the avoidance of burden. For example, older persons with a completed life perspective often rely on legal and cultural narratives of autonomy and control to legitimize their death wishes, even in the absence of clinical suffering (3). This dynamic is further illustrated in the Dutch and Belgian debates on ‘completed life’ (4). These discussions reveal a cultural ambivalence: while autonomy is valued, there is also discomfort with normalizing death wishes among non-terminally ill older people. As a result, societal expectations, legal norms, and institutional practices may reinforce or diminish them, depending on the sociocultural climate.

The interpretation of death wishes and end-of-life preferences is culturally and religiously embedded, and may differ substantially beyond Western liberal-autonomy frameworks. In several Buddhist ethical traditions, for example, principles of non-harm (ahimsa), impermanence, and acceptance of suffering and dying may shape attitudes toward ageing and death in ways that emphasize letting go rather than control or self-determination. Within palliative and spiritual care literature, such perspectives have been associated with meaning-making processes that prioritize acceptance, relational harmony, and existential integration at the end of life (45).

Emerging empirical work further suggests that cultural practices involving death contemplation may not be associated with distress or suicidality, but can instead relate to psychological well-being and acceptance. For instance, studies among older Buddhist meditation practitioners in Thailand (46) indicate that structured contemplative engagement with mortality may be linked to reduced fear of death and increased acceptance, highlighting that death-related cognitions are culturally patterned and context-dependent.

These cross-cultural perspectives suggest that constructs such as “completed life,” voluntariness, and appropriate clinical responses cannot be fully understood without considering underlying cultural and spiritual frameworks that shape their meaning and moral interpretation.

### Ethical and legal considerations

The increasing visibility of death wishes among older people in both public and medical domains has intensified societal and political debate on the societal and political debate on the legalization of MAID. This growing visibility might contribute to the expanding number of countries that are introducing legal frameworks for MAID, even in contexts where religious traditions-such as certain branches of Islam, orthodox Judaism and conservative Christian denominations – generally oppose assisted dying (47). However, legal regulation does not end the debate, as is evident from the Netherlands, the first country to legalize MAID. In the Netherlands, as one of the few countries, MAID is permitted for non-terminally ill people, including those with psychiatric disorders, dementia, and an accumulation of age-related conditions. Even in this context, the debate continues and is currently focused on MAID in adolescents with psychiatric disorder (48), older people with completed life (49), as well as interest in voluntary stopping with eating and drinking (4).

#### Legal frameworks and the expansion of MAID

After the Netherlands and Belgium have legalized MAID in 2002, more and more countries have legalized MAID (see https://www.IAADM.org). Nonetheless, only six countries have legalized MAID for dementia (Netherlands, Belgium, Luxembourg, Spain, Colombia, Canada, and Switzerland), and five of them (excluding Canada), also for psychiatric disorders.

In countries where MAID is legally available for psychiatric patients, empirical studies suggest that the due care criteria—particularly regarding decision-making capacity, irremediability of suffering, and the voluntariness of the request—are far more difficult to assess and operationalize in psychiatric contexts (5). Psychiatric conditions often lack objective markers of prognosis or irremediability, making it inherently difficult to determine whether suffering is truly without prospect (50, 51). Moreover, fluctuating decisional capacity, ambivalence, and comorbid personality traits further complicate assessments (52, 53). Recent evidence suggests that many psychiatric patients who initiate a MAID - trajectory experience ambivalence, and a substantial proportion eventually withdraw their request (49, 53). This underscores the dynamic nature of psychiatric suffering and challenges assumptions about the stability of death wishes in this population.

Additionally, people with dementia generally fear the prospect of being bereaved of their personality and dignity and prefer a timely death over having to live through the progressive stages of dementia (54). These fear might (partly) explain why most dementia cases have been given MAID in the early stages of dementia (55, 56). Nonetheless, in the Netherlands MAID may be provided for advanced dementia when legal criteria are met at the time of the (advanced) request (56), while in Canada which permits MAID under Bill C-7 for serious conditions, advance requests for MAID in dementia remain legally unresolved. This nuanced legal context has contributed to polarized political and societal debates regarding MAID in cases of advanced cognitive decline (57, 58).

In response, a care ethics perspective has been advanced, emphasizing relational autonomy and the importance of sustained therapeutic engagement over a purely rights-based or autonomy-driven model (59). Moreover, especially among older adult’s death wishes may arise from a combination of geriatric syndromes, multiple chronic diseases and subthreshold psychiatric disorders of which each would not qualify as a ground for MAID but in interaction drives their death wish (1). These developments have sparked international debate and raise complex questions about autonomy, vulnerability and the limits of medical intervention.

#### The “Completed Life” debate

The concept of a “completed life” has become emblematic of a new frontier in MAID ethics. It refers to a death wish independent of specific medical conditions, but on the subjective perception that one’s life is complete, purposeless, or devoid of future value. Among 1,563 Dutch middle-aged and older adults (≥57 years), 62 (4.0%) expressed a current death wish or weak wish to live of which only 11 (0.7%) had a moderate to strong death wish (3). Examination of these 62 participants by a licensed SCEN-physician (SCEN, Support and Consultation of Euthanasia in the Netherlands) revealed that only 6 individuals had neither a medically classifiable condition nor accumulation of age-related health problems that is needed to receive MAID (3). Among 21,294 Dutch citizens aged 55+, 267 (1.25%) reported a persistent death wish and no severe illness, based on their own perception of health (17). Of this group, 36 (0.17%) individuals had a wish to actually end their lives, of which 13 (0.06%) would like assistance (17). While the term ‘completed life’ has a positive connotation, the narrative of these individuals generally show a negative spiral of increasing problems, including loss of social connectedness, perceived loss of control over one’s life, financial difficulties, a reduced social network, relational problems, loneliness, experiences of loss, traumatic life events and adversity, depressive symptoms, physical decline, consequences of ageing and illness, dependency, and excessive worrying or rumination (1). Although the association between ‘completed life’ and psychiatric disorders has not yet been systematically studied, these correlates of ‘completed life’ may suggest that many individuals with a sense of completed life might have undiagnosed psychiatric disorders (17, 59).

Although Swiss law does not require the presence of a medical or psychiatric condition for MAID, right-to-die organizations apply strict eligibility criteria in practice, making it virtually impossible to access MAID solely on the basis of a ‘completed life’ or other non-medical reasons (60). The Dutch government considered, but ultimately postponed, legislation specifically addressing completed life requests outside the current MAID law. Main arguments given by the advisory committee was the difficulty to reliably operationalize criteria for completed life as well as sufficient room in the current Dutch legislation to perform MAID based on an accumulation of age-related conditions (which would apply for most people with ‘completed life’) (49). Nevertheless, this debate has ignited strong ethical controversy. Proponents argue that respect for autonomy entails recognizing individuals’ right to determine the endpoint of their life narrative, especially in the absence of dependence on others. Critics warn, however, that institutionalizing such requests risks normalizing death as a response to social or existential suffering, thereby potentially undermining social solidarity and the value placed on ageing lives. Specific concerns have been raised about implicit ageism and the risk of subtle societal pressure on vulnerable older adults to perceive themselves as expendable. Some death wishes may reflect internalized social devaluation rather than a truly autonomous choice (19, 44). In this view, broadening the legal and moral grounds for MAID without addressing the underlying social determinants of death wishes may produce ethically untenable outcomes.

### Clinical practice and communication

The clinical encounter with an older adult who expresses a wish to die presents a significant challenge for healthcare professionals. Such expressions often evoke ambivalence, moral unease, and diagnostic uncertainty among clinicians, especially when they are not clearly associated with a psychiatric disorder or poor somatic health. These situations may reveal structural gaps in care provision, as well as a lack of training and institutional guidance for navigating existential concerns in geriatric populations (23, 61).

#### Barriers to open dialogue

One of the most consistent findings is the reluctance of both patients and professionals to initiate conversations about death wishes. In a qualitative study, general practitioners felt ill-equipped to address death wishes unless they were clearly linked to depressive symptoms or suicidal risk (62). The fear of ‘opening Pandora’s box’ was frequently cited, reflecting concerns about unintentionally legitimizing or reinforcing such wishes through discussion. Older adults themselves often hesitate to disclose their thoughts due to fear of involuntary psychiatric interventions, stigmatization, or not being taken seriously (35). The fear of ‘opening Pandora’s box’ was frequently cited, reflecting concerns about unintentionally legitimizing or reinforcing such wishes through discussion. Older adults themselves often hesitate to disclose their thoughts due to fear of involuntary psychiatric interventions, stigmatization, or not being taken seriously (43).

#### Interpretive complexity and the risk of reductionism

Another key challenge is the risk of reductionism in clinical interpretation. Death wishes are frequently reduced to symptoms of mood disorders or suicidality. While such an approach may be warranted in some cases, it risks overlooking the existential, relational, and narrative dimensions of the wish (30). Particularly in older patients with multimorbidity, cognitive decline, or social isolation, such wishes may reflect a cumulative loss of identity, agency, and social worth rather than an acute psychiatric crisis. This underscores the need for interpretive competence; the clinician’s ability to navigate between medical frameworks and existential meanings. This may require more than clinical training: it demands ethical sensitivity, narrative literacy, and time to build relationships of trust (2).

#### Towards palliative and relational approaches

Given the complex etiology of death wishes, several authors have advocated for integrating palliative care principles into the management of such cases, even in the absence of terminal illness. A palliative approach emphasizes symptom control, existential support, and especially when death is not foreseeable in the near future, the co-construction of meaning in life. Healthcare professionals who adopted such a stance were better able to contain and respond to death wishes without either pathologizing them or acceding prematurely to requests for assisted dying (62–64). Moreover, relational approaches, such as those derived from care ethics, stress the importance of mutual engagement, sustained dialogue, and contextual understanding. This may not only alleviate the wish to die but also restore a sense of human dignity and narrative agency. In clinical practice, this entails a shift from ‘fixing’ to ‘witnessing’ and from problem-solving to accompaniment. Structured interventions, such as dignity therapy or meaning-centered psychotherapy [e.g. 64], might offer valuable tools but merits empirical evaluation in older populations. Nonetheless, even in palliative care, physicians often find it difficult to move beyond the medical-therapeutic perspective and to address existential concerns (65).

## Discussion

### Multidimensional nature of death wishes

The findings reviewed in this synthesis challenge reductive interpretations of death wishes in older adults as mere expressions of psychiatric morbidity or medical futility. A multidimensional perspective is required, situating such wishes within the broader contexts of biography, identity, relational embeddedness, existential meaning, and structural-cultural influences (7). While depression and hopelessness remain salient correlates (30), many older adults articulate death wishes in the absence of psychopathology, framing them instead as rational responses to cumulative losses in autonomy, agency, and social worth (9). This aligns with a growing body of literature emphasizing that death wishes often reflect existential distress, social marginalization, or narrative disruption, rather than purely clinical phenomena (20).

### Dynamic and relational dimensions

A critical finding is the dynamic and relational nature of death wishes. They are rarely static statements; rather, they fluctuate depending on interpersonal interactions, changes in physical or cognitive health, and shifts in social or existential context (30, 66). In psychiatric contexts, many patients withdraw their MAID requests over time (50, 53). Patients with neurodegenerative conditions may postpone MAID requests repeatedly, eventually losing decisional capacity, illustrating the ethical and practical challenges of balancing autonomy with protective care (55, 56). Longitudinal studies show death wishes fluctuate with health, social connectedness, and meaning-making, underscoring the limitations of purely autonomy-focused or clinical reductionist interpretations (43).

### Ethical dimensions

Ethical reflection is central to understanding and responding to death wishes. While respect for autonomy is fundamental, the ambivalent and context-dependent nature of these wishes challenges a purely autonomy-driven approach. Older adults may be influenced by internalized ageism, social marginalization, or perceived burdensomeness, raising questions about whether their choices reflect genuine free will (67). Care ethics emphasizes relational and societal responsibility, highlighting the clinician’s duty to engage in dialogue while addressing structural conditions that exacerbate existential suffering (68). Virtue ethics complements this by stressing moral qualities such as empathy, patience, and courage in navigating MAID requests (69).

From a policy and societal standpoint, frameworks allowing assisted dying for non-terminal conditions require safeguards against coercion or social pressure, promoting social inclusion and equitable access to psychosocial support (49). Integrating these ethical considerations into clinical training—through simulation, case discussions, and reflective supervision—can cultivate nuanced judgment while respecting patient dignity. Ethics-informed training is especially important for addressing ambivalence, relational autonomy, and social inequities, ensuring that clinicians are prepared to navigate complex moral dilemmas (70).

### Clinical implications and training

Recognizing the relational and existential dimensions of death wishes is essential in clinical practice. Rather than interpreting such wishes solely as symptoms of psychopathology or as direct requests for assisted dying, clinicians should approach them as expressions of complex experiences of suffering, including existential distress, loneliness, perceived burdensomeness, or loss of meaning. Palliative and dignity-enhancing approaches can address these concerns without prematurely framing them in terms of MAID eligibility (71, 72). Narrative and relational interventions allow clinicians to explore the personal and social contexts in which death wishes emerge, facilitating the co-construction of meaning and identification of modifiable sources of distress such as social isolation or loss of dignity (73).

Clinical responses are often constrained by epistemic uncertainty, arising from the complex interaction of psychological, social, existential, and legal factors – underscoring the need for interdisciplinary collaboration and societal support. Assessing the meaning and persistence of a death wish can therefore be challenging, particularly when psychiatric pathology is absent or ambiguous (74). This uncertainty underscores the importance of interdisciplinary collaboration involving psychiatry, geriatrics, palliative care, ethics consultation, and social services, alongside broader societal support structures addressing loneliness, marginalization, and existential suffering.

Improving professional preparedness is therefore crucial. Training programs should extend beyond biomedical frameworks and incorporate simulation-based education, interprofessional case discussions, and reflective supervision, enabling clinicians to practice navigating ethically and emotionally complex scenarios (75). Greater integration of palliative care principles, existential care, and ethics education within geriatric and mental health training may strengthen clinicians’ ability to respond constructively to death wishes (76). Interventions such as dignity therapy, meaning-centered approaches, and narrative medicine provide practical tools to engage with existential suffering while respecting patient autonomy and dignity (77).

Such approaches also have preventive value, as early recognition and engagement with existential distress can reduce the risk of persistent death wishes or requests for assisted dying (78). Through sustained dialogue, ethical reflection, and interdisciplinary support, clinicians not only facilitate ethically sound decision-making but also preserve dignity, meaning, and relational connectedness in later life (68). These clinical efforts naturally link to broader sociocultural and policy considerations, emphasizing that individual care must be embedded within social structures that promote inclusion, counter ageism, and provide equitable psychosocial support.

### Sociocultural and structural influences

Sociocultural dynamics—including ageism, social exclusion, and internalized societal devaluation—must be explicitly addressed in both clinical practice and policy (79, 80). Clinicians can implement structured psychosocial and narrative assessments exploring social inclusion, perceived burdensomeness, and existential meaning (81). Interventions may include community engagement programs, peer-support networks, guided meaning-making, or dignity therapy sessions aimed at restoring agency and relational embeddedness (82).

From a policy perspective, public health initiatives targeting loneliness, social integration, and access to psychosocial support can reduce structural vulnerabilities that might otherwise legitimize death wishes as socially acceptable responses to marginalization (83). MAID legislation should explicitly consider these determinants to ensure that requests reflect genuine autonomy rather than societal neglect or pressures. Policies promoting meaningful participation of older adults, alongside campaigns countering ageist narratives, can rehumanize life amidst decline and prevent structurally induced suffering (79).

### Research directions

Research gaps remain substantial. Few longitudinal studies examine the evolution of death wishes over time, and few interventions aim to understand rather than extinguish these wishes (78, 83). Future research should adopt mixed-method and participatory designs, focusing on diversity in gender, culture, and socioeconomic status, and considering social and existential determinants. Evaluating the impact of ethics-informed training, interprofessional case discussions, simulation-based education, and palliative integration on clinician competence and patient outcomes is critical.

## Conclusion

Death wishes in later life constitute a complex, multidimensional phenomenon that transcends the boundaries of psychiatry, gerontology, and ethics. They emerge at the intersection of psychological vulnerability, existential suffering, and sociocultural marginalization. While depression and demoralization may contribute, many wishes to die are more fundamentally rooted in a perceived loss of narrative coherence, meaning, and social embeddedness.

Such wishes are not merely individual or clinical in nature, but relational and contextually situated. Accordingly, adequate responses must integrate clinical, ethical, existential, and policy perspectives, with careful attention to sociocultural determinants. This includes strengthening ethical and narrative competence among professionals, as well as fostering social and relational inclusion.

Clinical experience suggests that sustained engagement, reflective practice, and interdisciplinary collaboration are often decisive in supporting older adults to rediscover meaning or reconsider death wishes, even in highly complex cases. At the same time, structural inequities that shape vulnerability in later life must be explicitly recognized and addressed.

Policy and legal frameworks, including Medical Assistance in Dying (MAID), require not only rigorous safeguards but also critical awareness of the broader societal narratives they reflect and help to shape. This calls for an approach that moves beyond both diagnostic reductionism and an uncritical emphasis on individual autonomy, and instead foregrounds relationality, interpretive depth, and existential responsiveness.

In ageing societies, the central ethical imperative may not lie primarily in enabling or preventing death, but in rehumanizing life in the face of decline. By promoting dignity, autonomy, and inclusion, healthcare systems can contribute to this rehumanization (84). Understanding and responding to death wishes, therefore, is not solely a clinical task, but a shared societal responsibility.
